# Specific IgE Anti-Ascaris in Brazilian Children and Adolescents

**DOI:** 10.1097/WOX.0b013e3181d4c2dd

**Published:** 2010-03-15

**Authors:** Emanuel S Sarinho, Décio Medeiros, Almerinda Silva, José Ângelo Rizzo

**Affiliations:** 1 Federal University of Pernambuco, Recife, Brazil

**Keywords:** ascaris, anti-ascaris IgE, geohelminths, allergy, asthma

## Abstract

From an article published by our group by Medeiros et al, we discuss and review the literature on the role of serum specific anti-ascaris IgE in patients with respiratory allergies living in countries where helminthic infestations are common. Medeiros et al conducted a study using 101 patients aged 12 to 21 years with respiratory allergy. Median IgE level was 660 IU/mL. Serum specific anti-ascaris IgE was positive in 73% (74/101) of the individuals, but parasitological stool examination yielded positive results in only 33.7% (34/101). The correlation coefficient between serum total IgE level and serum specific anti-ascaris IgE was 0.52 (*P *< 0.001) and this effect occurred regardless of eosinophil count and of the presence of intestinal helminthic infection. In patients with respiratory allergy with very high serum total IgE levels, in whom the past or present history of parasitic infection is a possible explanation, the presence of serum specific anti-ascaris IgE was common and should be better evaluated in allergic patients from *Ascaris lumbricoides *endemic areas.

## Introduction

Heminthic infections are frequent in Latin America countries especially in rural areas but are also common in low income families living in urban centers. Allergic diseases are an important public health problem worldwide and bigger cities in Brazil, asthma is reported with a high prevalence [1]. The interaction between parasite infections and allergy may mean epidemiological evidence that challenges the hygiene hypothesis.

A serum total IgE level >200 IU/mL in individuals from developed countries with family history of atopy suggests a possible development of allergic disease in the future [2]. Nevertheless, several factors have an impact on serum total IgE levels, as IgE is not only an expression of atopy. High concentrations of serum total IgE can also be observed in some infections, such as HIV, chronic hepatitis, and rare primary immunodeficiency with recurrent infections. Despite that, serum total IgE high levels in patients from poor countries frequently indicate chronic parasitic infection, especially by geohelminths, which stimulate the production of polyclonal IgE [3,4].

*Ascaris lumbricoides *tend to cause chronic infections, which can interfere with T_H_2 response. It is possible that this mechanism enables parasite survival and may allow the host to escape from potentially damaging inflammation in the tissues. The relationship between this geohelminth and allergy can be manifested in different ways according to time of first infection, intensity of infection, and host genetic background [5]. Early and chronic infections tend to suppress allergic inflammation. Light infections with this parasite may increase the allergic response in predisposed individuals [5]. Furthermore, increased serum total IgE levels in a patient with respiratory allergy can be influenced by intestinal parasites, especially by *A. lumbricoides*, the most prevalent geohelminth in Brazil. Therefore, Medeiros et al [6] from our group conducted a study to assess the relationship between total blood IgE levels, eosinophil counts, and serum anti-ascaris specific IgE levels in adolescents with asthma and/or allergic rhinitis. The cross-sectional study was carried out at the Research Center for Allergy and Immunology at Hospital das Clínicas of Recife, affiliated to Universidade Federal de Pernambuco-Brazil. Patients with asthma and/or allergic rhinitis aged 12 to 21 years were selected. Respiratory allergy was diagnosed in those patients with asthma and/or allergic rhinitis with serum total IgE levels greater than those expected for age, with serum specific IgE to aeroallergens >0.35 IU/dL and/or with skin tests showing immediate hypersensitivity to mites >3 mm (compared with the negative control) [7,8].

All patients were referred to the allergy center because of their sufficiently remarkable symptoms to make them seek the city's referral center for allergy. After anamnesis and physical examination, blood and stool samples were collected from patients. Serum total IgE level was measured by the Phadia UniCAP System (Phadia Upjohn, Uppsala, Sweden), and a complete blood count was performed to determine the number of eosinophils. Presence of intestinal parasites was assessed by the examination of fresh stool specimens using Hoffman and Baermann-Moraes methods. Serum specific IgE to *A. lumbricoides *was quantified by the enzyme immunoassay technique using Phadia UniCAP System (Phadia Upjohn).

In this study, the sample consisted of 101 patients, 56% (57/101) of whom were male and the mean age was 14.9 ± 2.36 years. With regard to respiratory allergy, asthma was diagnosed in 24% (25/101) of patients, whereas 33.7% (34/101) had allergic rhinitis and 41.5% (42/101) showed coexistence of asthma and allergic rhinitis. The parasitological stool examination yielded positive results for geohelminths in 33.7% (34/101), and *A. lumbricoides *was the most prevalent parasite, with a rate of 47% (16/34), followed by *Trichuris trichiura*, with 35.3% (12/34).

Table 1 shows the total serum IgE and serum specific anti-ascaris IgE levels and the eosinophil count. The correlation coefficients between serum total IgE and eosinophil count, serum total IgE and serum specific anti-ascaris IgE, and eosinophil count and serum specific anti-ascaris IgE were 0.34 (*P *= 0.001), 0.52 (*P *< 0.001), and 0.26 (*P *= 0.01), respectively. Table 2 shows the final multiple linear regression model. Serum specific anti-ascaris IgE contributed to the total serum IgE, and this effect occurred regardless of eosinophil count and of the presence of intestinal helminthic infection. The coefficient of determination (adjusted *R*^2^) was 0.25 (*F *= 12.35; *P *< 0.001).

**Table 1:** Median Levels of Total IgE, Anti-Ascaris IgE, and Eosinophil Count in 101 Adolescents With Asthma and/or Allergic Rhinitis

Variables	Median	P_25-75_
Total IgE (UI/mL)	660	243.5-1500
Anti-ascaris IgE (IU/mL)	2.1	0-5.2
Eosinophils (mm^3^)	510	284-811

Adapted from Medeiros D, Silva AR, Rizzo JA, Motta ME, Oliveira FH, Sarinho ES. Total IgE level in respiratory allergy: study of patients at high risk for helminthic infection. *J Pediatr (Rio J)*. 2006;82:255-259.

**Table 2:** Final Multiple Linear Regression Model for Total Serum IgE (IU/mL) in 101 AdolescEnts With Asthma and/or Allergic Rhinitis

Variables	*β *(CI)	*F *statistics	*P*
Anti-ascaris IgE (IU/mL)	4.17 (2.45, 7.08)	29.09	< 0.001
Eosinophils (mm^3^)	1.41 (0.85, 2.40)	1.83	0.15
Intestinal parasitic infection	0.08 (-0.12, 0.29)	0.63	0.34

Adapted from Medeiros D, Silva AR, Rizzo JA, Motta ME, Oliveira FH, Sarinho ES. Total IgE level in respiratory allergy: study of patients at high risk for helminthic infection. *J Pediatr (Rio J)*. 2006;82:255-259.

CI, confidence interval.

## Specific serum specific anti-ascaris ige in allergic respiratory patients

Elevated serum total IgE levels and eosinophils are not only associated with allergies but also with helminthic infections, [9,10] and this is why we chose to assess this relationship in our study [6]. We found that 49.5% (50/101) of patients with respiratory allergy had eosinophil counts within the normal range suggesting the participation of other cells in the allergic inflammatory process [11]. However, it should be recalled that the absence of peripheral eosinophilia does not rule out the possibility of lung tissue eosinophilic inflammation [11,12]. Serum eosinophils are more dependent on interleukin-5 (IL-5) for their maturation and survival, whereas tissue eosinophils are more responsive to the granulocyte and macrophage colony-stimulating factor (GM-CSF) originating from innate immunity or derived from the T_H_1 axis [12]. Furthermore, eosinophil count can be normal in peripheral blood but active and filled with active granules with major basic protein, eosinophil cationic protein, and other mediators [12,13]. Another explanation for normal serum eosinophil count in 50% of these patients could be the presence of a mild inflammatory process, without any effects at the peripheral blood level, which may result from the use of medications to control the allergy or from the down-regulation of the number of eosinophils in circulating blood, and is a common finding in chronic intestinal parasitic infections [14]. After the migration of intestinal larvae, there is a remarkable reduction in eotaxin, which is essential for chemotaxis and eosinophilia in these patients [15].

The role of serum total IgE in the severity or persistence of bronchial hyper reactivity must be reassessed. Currently, serum total IgE level is a weak indicator of allergic respiratory disease [3]. Very high total serum IgE levels can result from polyclonal activation because of chronic infection by geohelminths,[16] which can probably confirm the hypothesis that high burden of intestinal parasitic infection can prevent atopy or reduce its severity [16-18]. Individuals from areas where *A. lumbricoides *is endemic show remarkable expression of T_H_2 cytokines [19]. Infected atopic individuals from these areas show exacerbation of allergic symptoms immediately after antihelminthic therapy. An adequate and chronic parasitic infection, mainly early in life, can modulate the immune response and reduce allergic inflammation and its pathophysiological effects. Another possibility to explain very high levels of serum IgE is that these individuals present a strong genetic predisposition to atopy, who are more likely to be hypersensitive to environmental allergens, also have stronger resistance to geohelminthic infections [16]. Copper et al [16] have shown that geohelminths can stimulate specific antibody production but also an exaggerated nonspecific polyclonal IgE synthesis. Consequently, these parasites are associated with high circulating levels of serum total IgE, which is an escape mechanism, that may result in the saturation of mast cells Fcξ IgE receptors, in the inhibition of allergenic reactivity and also of any defense action of specific IgE against the parasite [20].

The time at which parasitic infection occurs, the possibility of scarce parasite load or of larval forms, or frequent and repeated antihelminthic therapy should be taken into consideration when interpreting stool examination for parasites,[21] and its sensitivity in some cases can be as low as 40% [19]. In the study by Medeiros et al, positivity was around 40% and did not influence the total serum levels of IgE. By assessing intestinal parasitic infections and the levels of serum IgE in atopic and nonatopic patients, Nyan [21] could not find any relationship between parasitic disease and increased levels of serum total IgE. On the other hand, when analyzing serum specific antiascaris IgE in these patients, it was observed that it contributed to explain up to 25% of serum IgE levels. Dold et al [22] also observed that the presence of serum specific anti-ascaris IgE was associated with high levels of total serum IgE, even though they did not investigate parasitic infection.

Tropomyosin has been identified as a contractile protein that may act as a panallergen and might cause cross-reactivity between mites, cockroaches, shrimp, mollusks, and intestinal parasites, including *A. lumbricoides*. Therefore, such possibility should not be overlooked [9,10]. However, the influence of this protein on cross-reactions with serum specific anti-ascaris IgE still needs to be further investigated. Furthermore, there was a high prevalence of ascariasis in the analyzed sample in our research, and therefore, serum specific anti-ascaris IgE must represent hypersensitivity of these patients to recent or past infection by *A. lumbricoides*. Recently, some reports have shown that IgE and specific antiparasite IgG4 are markers of resistance and susceptibility of humans to ascariasis [23].

Immune and allergenic responses to intestinal parasitic infections vary and can be either acute or chronic. Usually, there is a specific response to the parasite in the acute phase, characterized by pronounced eosinophilia and high levels of specific IgE [24]. On this occasion, allergic syndromes can be observed, such as urticaria-like rashes or episodes of bronchospasm caused by larval migration across the lung tissue. At this stage, serum and tissue eosinophilia occur, in an attempt to deter or even eliminate the parasite. Thus, the high level of specific IgE plays an important role in increasing eosinophil recruitment to the site of invasion. Serum polyclonal IgE levels will be extremely high only at a later stage, after the host has been infected several times,[24] when the relationship between the host and the parasite seemingly reaches a plateau, however, parasitic infection does not cease to exist, but larval migration is reduced to a minimum. At this stage, enough and prolonged load of helminths may induce suppressor T cells and contribute to reduce allergic responses toward environmental allergens, to a remarkable production of IL-10 and of transforming growth factor beta (TGF-*β*), which, in its turn, reduce the number of serum eosinophils [24-26]. Lima et al [15] studied mice chronically infected by *A. suum *and observed a reduction in eosinophil count and in eosinophil peroxidase in the airways, and a marked reduction in IL-4 and IL-5 in the bronchoalveolar lavage.

Since 1987, very high levels of serum specific anti-ascaris IgE have been found in allergic patients in tropical regions [27]. In nonatopic patients, serum specific anti-ascaris IgE was negatively correlated with increased levels of serum total IgE,[28] which suggests that polyclonal IgE response against helminths is characteristic of atopic individuals. In children with predisposition to atopy, IgE response was associated with a protective response against helminths. The relationship between serum specific anti-ascaris IgE and serum total IgE was up to 9 times greater in atopic children with helminthic infection than in those children with parasitic infection but without allergy, which supports the hypothesis that predisposition to atopy can be a selective evolutionary advantage [29].

**Figure 1:**
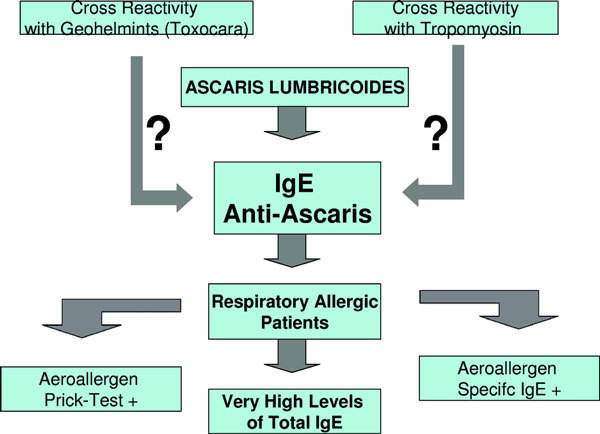
**Model explaining the possible role of anti-ascaris IgE in patients with respiratory allergy**.

Some studies have provided evidence for positive associations between the presence of anti-ascaris specific IgE and allergen skin test reactivity or elevated allergen-specific IgE and asthmatic symptoms [30,31]. Our study showed that anti-ascaris specific IgE was often positive and associated with high levels of serum total IgE in respiratory allergic patients. Anti-ascaris IgE may be an expression of the response of the atopic phenotype against the parasite, may be the result of cross reaction with other parasites such as toxocara or response to tropomyosin. According to Santos et al [32] cross-reactivity between *A. lumbricoides *and American cockroach tropomyosin is probably not clinically important. A proposal for a model explaining the role of serum specific anti-ascaris IgE in patients with respiratory allergy is suggested in Figure 1.

There is reasonable evidence that helminths and other infectious agents can induce nonspecific proliferation of activated B cells, resulting in very high levels of serum total IgE, which must be frequent in poor countries and in under-nourished populations [33]. The immune response to intestinal parasitic infection is initially characterized by the production of specific IgE followed by the increased synthesis of polyclonal IgE. However, the behavior of serum specific anti-ascaris IgE in the clinical context of specific diagnosis of ascariasis has not been sufficiently explored. Our work [6] is an observational study, the presented conclusions are merely speculative and additional studies for a better understanding of this specific IgE should be carried on.
